# Japanese Health Insurance Coverage of Fertility Treatment in 2022: Does Coverage Change Patient Perspectives?

**DOI:** 10.7759/cureus.74459

**Published:** 2024-11-25

**Authors:** Tsukasa Yoshida, Iwaho Kikuchi, Yusuke Sako, Takamitsu Kitano, Tetsuya Hirata

**Affiliations:** 1 Department of Obstetrics and Gynecology, St. Luke’s International Hospital, Tokyo, JPN; 2 Fertility Treatment, Medical Park Yokohama Clinic, Tokyo, JPN

**Keywords:** financial stress, infertility, insurance, in vitro fertilization, reproductive techniques

## Abstract

Objective: This study aimed to evaluate the change in the patient’s background and attitude toward infertility treatment both before and after the initiation of insurance coverage and to explore future issues from the patients’ perspectives.

Materials and methods: A cross-sectional survey was conducted in a fertility clinic in Japan from February to June 2022. An original questionnaire was given for two groups of new patients at a fertility clinic on their first visit: before fertility treatment insurance coverage started (Before-coverage) and after fertility treatment insurance coverage started (After-coverage).

Results: The study included 75 patients (Before-coverage = 25; After-coverage = 50). Multivariate analysis revealed increases among patients who consider infertility a “disease” rather than a “condition” (odds ratio (OR): 5.03, p < 0.05), those preferring in vitro fertilization (IVF) as an initial treatment (OR: 2.54, p = 0.03), and those who recommend oocyte freezing for one’s child (OR: 3.88, p = 0.04), with statistical significance in the After-coverage group compared with the Before-coverage group. However, the anticipated financial burden did not change significantly (e.g., cost per IVF, cost to achieve pregnancy).

Conclusions: More patients had an impression of infertility as a “disease” and preferred IVF as the first treatment option after coverage than before coverage. Furthermore, many patients wanted to recommend oocyte freezing for their children despite the lack of insurance coverage. However, patients’ anticipated economic burden for treatment was not different between both groups. The economic burden anticipated by patients remained unchanged, revealing the challenge of disseminating information to patients in the future.

## Introduction

The birth rate in Japan is declining annually [1]. As women participate in the workforce, they marry later in life, which increases the demand for assisted reproductive technology (ART). The ART birthrate has increased to 5% of all births in Japan [2]. To encourage the use of ART, insurance coverage for fertility treatment started to become available in Japan in April 2022. The foundation of the Japanese healthcare system lies in Kokumin-Kaihoken, a nationwide universal health coverage public insurance system [3], which provides that all women seeking fertility treatment will be covered by insurance.

Before insurance coverage began, the Ministry of Health, Labour and Welfare in Japan provided subsidies for ART to reduce the economic and psychological burden on infertile couples from 2004 to 2022 [4]. The eligibility criteria of the subsidy system had income restriction and age restriction as under 43 years old. The amount of subsidy was fixed regardless of treatment contents [5]. The subsidy program had some problems; there were cost discrepancies among facilities because the cost was not determined by the insurance system. While it had advantages, like the subsidy being paid regardless of the ART contents, which was helpful for patients with severe infertility needs, the treatment for infertility is not covered by the current insurance system [6].

Meanwhile, in the current insurance system, there is age restriction, but income restriction was eliminated. Furthermore, infertility patients can receive infertility treatment at a uniform nationwide reasonable rate. However, certain disadvantages have also been noted; the development of infertility treatment in Japan could be hampered due to the restriction of treatment options [6].

Since this is the first time that insurance coverage for fertility treatment has been implemented, the advantages and disadvantages of insurance coverage for patients are unclear.

Medical providers have an obligation to provide accurate information to alleviate patient concerns. However, little information is available about what patients want to know. Therefore, the purpose of this study was to evaluate the changes in the patient’s background, treatment-related knowledge, emotions, and objectives during their first visit both before and after the initiation of insurance coverage and to understand what patients want to know.

## Materials and methods

The study period was from February to June 2022. The study population comprised first-time patients and was divided into two groups: “before the start of fertility treatment insurance coverage start” (Before-coverage) group and “after the start of fertility treatment insurance coverage start” (After-coverage) group. We designed an anonymous questionnaire with multiple choices (Appendix) and conducted a cross-sectional survey at Medical Park Yokohama fertility clinic in Yokohama, Japan (an urban area near Tokyo). We recruited all patients at the clinic for our community-based survey by obtaining their consent to participate after explaining the study's purpose and assuring them that their anonymity would be maintained. All participants were asked to complete the questionnaire before their first appointment.

The primary endpoints of this study included differences in patient backgrounds (Q0-Q3; age, household income, and declaration to visit the clinic), treatment-related knowledge (Q4-Q6), expression of infertility (Q7), emotion during first-time visit (Q8), objectives during first-time visit (Q9; first treatment option, Q10; recommendations for oocyte freezing, and anticipated cost burden (Q11-13)). The secondary endpoints included whether the patients would answer open-type questions and whether their answers to these questions would include a positive expression of insurance coverage (e.g., expect, happy, and so on). The reason why we set the question related to oocyte freezing (Q10), even though it is not covered by insurance, was due to the fact that we anticipated the growth in knowledge about infertility treatment in Japan, which would lead to an increased awareness of the importance of retrieving oocyte at a young age for conception.

Mann-Whitney U test and Fisher’s exact test were performed as univariate analysis in Q0-Q3. Regarding multivariate analysis, multiple logistic regression analysis including Q1 as a confounder was conducted in Q4-Q13. R (version 3.5.0) and EZR (The R Foundation for Statistical Computing, Vienna, Austria) were used for all the statistical analyses [7]. This study was approved by the Institutional Ethics Committee of Medical Park Yokohama Clinic (registration number, 20220222-1) and complied with the requirements under the Declaration of Helsinki. Informed consent was obtained through the opt-out form on the website.

## Results

Seventy-five patients were included (median age, 33 years): the Before-coverage group (25 patients, 33%) and the After-coverage group (50 patients, 67%). The response rate to this survey was 100% (n = 75). In total, 24% (n = 18) of the patients described their emotions at the first-time visit as “depressed” (Q8), and none of the patients reported that the cost of in vitro fertilization (IVF) was “inexpensive” in the question about anticipated cost burdens (Q13). Univariate analysis revealed no statistically significant changes in the background factors: age at visit (Q0), history of visits to other fertility clinics before (Q1), household income (Q2), and declaration to visit clinic (Q3) (Table 1).

**Table 1:** Univariate analysis outcome (Q0-Q3) †Data are presented as median (range) for continuous variables. ‡Data are presented as number (%) for categorical variables. * p-value <0.05. After-coverage, after the commencement of fertility treatment insurance coverage; Before-coverage, before the commencement of fertility treatment insurance coverage group.

Variables		Overall (n = 75)	Before-coverage (n = 25)	After-coverage (n = 50)	p-value
Q0: Age^†^, years		33 (25-85)	33 (26-48)	34 (26-46)	0.322
Q1: History of visits to other infertility clinics before^‡ ^	First-time visit	45 (60%)	17 (68%)	28 (36%)	0.384
	Examination/consultation only	17 (23%)	4(16%)	13 (26%)	
	Visit with treatment	13 (17%)	4 (16%)	9 (18%)	
Q2: Annual household income^†^, thousand yen		900 (300-1200)	900 (300-12000)	900 (3-1200)	0.410
Q3: Told colleagues in your workplace about this visit^‡^	Not plan to do	47 (64%)	20 (80%)	27 (55%)	0.054
	Plan to do	8 (11%)	0 (0%)	8 (16%)	
	Told	19 (26%)	5 (20%)	14 (29%)	

According to the multivariate logistic regression analysis, the number of patients who considered infertility a “disease” rather than a “condition” was statistically significantly higher in the After-coverage group compared to the Before-coverage group (odds ratio (OR): 5.03, 95% confidence interval (CI) (1.040-24.40), p < 0.05: Q7). Furthermore, those who considered IVF an initial treatment (OR: 1.52, 95% CI (1.030-2.24), p = 0.04: Q9) and those who wanted to recommend oocyte freezing for their children if they had a girl in the future (OR: 2.60, 95% CI (1.040-6.53), p = 0.04: Q10) were statistically significantly greater in the After-coverage group compared with the Before-coverage group (Table 2).

**Table 2:** Multivariate analysis outcome (Q4-Q13) †Data are presented as median (range) for continuous variables. ‡Data are presented as number (%) for categorical variables. §On a scale of 1 to 5, with 1 being “Do not know” and 5 being “Know”. ¶On a scale of 1 to 5, with 1 being “Depressed” and 5 being “Looking forward”. †† On a scale of 1 to 5, with 1 being “Not considering” and 5 being “Considering”. ‡‡On a scale of 1 to 5, with 1 being “Will not recommend” and 5 being “Would Recommend”. §§ On a scale of 1 to 5, with 1 being “Inexpensive” and 5 being “Expensive”. *p-value < 0.05. After-coverage, after the commencement of fertility treatment insurance coverage; Before-coverage, before the commencement of fertility treatment insurance coverage. ICSI, intracytoplasmic sperm injection; IUI, intrauterine insemination; IVF, in vitro fertilization.

Variables		Overall (n=75)	Before-coverage (n=25)	After-coverage (n=50)	p value
Q4: The knowledge on difference between IUI and IVF^‡^^§^	1	5 (7%)	3 (12%)	2 (4%)	0.150
	2	8 (11%)	2 (8%)	6 (12%)	
	3	16 (21%)	8 (32%)	8 (16%)	
	4	32 (43%)	9 (36%)	23 (46%)	
	5	14 (19%)	3 (12%)	11 (22%)	
Q5: The knowledge on the difference between IVF and ICSI^‡^^§^	1	14 (19%)	7 (28%)	7 (14%)	0.123
	2	20 (27%)	5 (20%)	15 (30%)	
	3	17 (23%)	9 (36%)	8 (16%)	
	4	15 (20%)	3 (12%)	12 (24%)	
	5	9 (12%)	1 (4%)	8 (16%)	
Q6: The knowledge on oocyte freezing^‡^^§^	1	4 (5%)	2 (8%)	2 (4%)	0.957
	2	12 (16%)	4 (16%)	8 (16%)	
	3	25 (33%)	5 (20%)	20 (40%)	
	4	22 (29%)	11 (44%)	11 (22%)	
	5	12 (16%)	3 (12%)	9 (18%)	
Q7: Expression of infertility^‡^	Condition	17 (23%)	22 (92%)	33 (69%)	0.045^*^
	Disease	55 (77%)	2 (8%)	15 (31%)	
Q8: Emotion during the first-time visit^‡^^¶^	1	3 (4%)	1 (4%)	2 (4%)	0.381
	2	15 (20%)	6 (24%)	9 (18%)	
	3	35 (47%)	12 (48%)	23 (46%)	
	4	20 (27%)	6 (24%)	14 (28%)	
	5	2 (3%)	0 (0%)	2 (4%)	
Q9: Consider IVF as the initial treatment ^‡ ††^	1	13 (17%)	6 (24%)	7 (14%)	0.036^*^
	2	17 (23%)	6 (24%)	11 (22%)	
	3	18 (24%)	8 (32%)	10 (20%)	
	4	13(17%)	5 (20%)	8 (16%)	
	5	14 (19%)	0 (0%)	14 (28%)	
Q10: Recommendation of oocyte freezing for your daughter^‡ ‡‡^	1	1 (2%)	1 (5%)	0 (0%)	0.042^*^
	2	2 (3%)	1 (5%)	1(2%)	
	3	44 (65%)	17 (77%)	27 (59%)	
	4	17 (25%)	2 (9%)	15 (33%)	
	5	4 (6%)	1 (5%)	3 (7%)	
Q11: Expectation of cost to perform one IVF procedure ^†^, thousand yen		30 (1-150)	30 (3-100)	30 (1-150)	0.500
Q12: Expectation to incur until achieving pregnancy ^†^, thousand yen		100 (10-400)	100 (10-400)	100 (10-300)	0.776
Q13: Impression of the costs in Q11 and Q12?^‡ §§^	1	0 (0%)	0 (0%)	0 (0%)	0.591
	2	0 (0%)	0 (0%)	0 (0%)	
	3	11 (17%)	5 (22%)	6 (14%)	
	4	46 (70%)	15 (65%)	31 (72%)	
	5	9 (14%)	3 (12%)	6 (14%)	

The response rate to the open question was 12% (n = 3) for the Before-coverage group and 20% (n = 10) for the After-coverage group (p = 0.40), and there were more comments indicating positive expectations from insurance coverage of fertility treatment in the After-coverage group than in the Before-coverage group (p = 0.11). However, no statistically significant difference was found between either groups for the open-ended questions on response rates and the rates of including positive expression of insurance coverage.

Moreover, no statistically significant changes were found for knowledge of treatment (Q4-Q6), feelings at a consultation (Q8), and anticipated cost burden (Q11-Q13) (Table 2).

## Discussion

This study had four implications. First, insurance coverage for fertility treatment was associated with an increase in patients who consider infertility a “disease” (in Q7). Second, those who considered IVF an initial treatment (Q9) and those who wanted to recommend oocyte freezing for their children if they had a girl in the future were statistically significantly greater (Q10) in the After-coverage group compared with the Before-coverage group. Third, there was no statistically significant difference between both groups for the anticipated cost burden of fertility treatment (Q11-Q13). To the best of our knowledge, this is the first report on the influence of insurance coverage on patients’ attitudes toward infertility and ART in Japan.

Regarding the expression of infertility, the Centers for Disease Control and Prevention (CDC) and World Health Organization (WHO) have argued for defining infertility as a disease of the reproductive system [8,9]. Moreover, a previous report showed that in the U.S., those who considered infertility a “disease” rather than a "condition" were more interested in fertility treatment (p = 0.014) and fertility preservation (p = 0.017), and they were more likely to use treatments covered by insurance [10]. Furthermore, the authors suggested that changing the patient’s perception of infertility from a “condition” to a “disease” will lead to an improved consultation rate for fertility treatment. Similarly, in Japan, insurance coverage led to the patient’s recognition of infertility as a “disease,” which may encourage people to undergo fertility treatment in the future.

As for the initial treatment, the preference for IVF was increased after the commencement of the insurance coverage. Reportedly, in Japan couples with lower household incomes were less likely to seek medical help for infertility than those with higher household incomes [11]. In terms of less financial burden, the decision to start IVF treatment is mostly influenced by insurance coverage [12]. There are also cases wherein IVF treatment is withdrawn before conception. The major reasons for women with infertility withdrawing from IVF treatment were reported to be excessive stress (40.2%), followed by financial pressures (25.1%) and loss of insurance (24.6%) [13]. As described above, the financial impact of insurance coverage on a patient’s willingness to begin or withdraw IVF treatment is significant. Insurance coverage has reduced the financial burden of IVF treatment in Japan, which may lower the hurdle of undergoing IVF treatment. However, there was no difference in the anticipated cost burden for undergoing IVF and the impression of these costs in our data. As per our speculation, the participants had an idea that the cost of IVF would become cheaper, but they did not have any idea or information regarding how much it would actually cost. This means that the patients did not receive information concerning the specific costs of IVF before coming to the clinic. It is thus necessary to provide accurate information on the financial burden of patients.

Although oocyte freezing was not included in the insurance coverage of this study, the ratio of patients who wanted to recommend the procedure to their children increased. Reportedly, the demand for fertility preservation is increasing in Japan where women’s empowerment is gradually becoming an essential part of the society [14]. However, many are still unable to undergo fertility preservation due to high financial burdens [14]. As social oocyte freezing, one study subsidized oocyte freezing for women who wanted fertility preservation for various reasons in Urayasu City in Japan [14]. The authors suggested that a subsidy could be a policy to solve the declining birth rate due to delayed marriage in Japan. The issue of fertility preservation is not only about cost but also about the low utilization rate of frozen eggs. Worldwide, the utilization rate of frozen oocytes is remarkably low, at 3.1-9.3% [15,16]. Because of the low utilization rate, some reviews concluded the low-cost effectiveness of social oocyte freezing, which reported the extra cost of each live birth between $600,000 and $1,000,000 [17]. If Japan implements social oocyte freezing, we will face issues related to its low cost-effectiveness. Insurance coverage for ART may have increased public awareness of the importance of fertility preservation; however, there are many problems yet to be solved in implementing social oocyte freezing in Japan.

In this study, we found no significant differences between both groups regarding the degree of knowledge about fertility treatment. Furthermore, according to the question on emotion in our data, about 30% of participants described their feeling as “depressed.” Prior reports indicated that more than half of infertile women who initiated IVF felt mild or worse depressive symptoms in Japan [18]. Moreover, women’s anxiety levels regarding fertility treatment were significantly higher before treatment than during treatment, and their anxiety stemmed from the lack of information and awareness regarding solutions to counter infertility [19]. Furthermore, fertility knowledge in Japan is reported to be remarkably low compared with other developed countries [20]. This is a problem that needs to be resolved. One cross-sectional survey concluded that the fertility knowledge of the Japanese population improved; however, it was still low, and thus educational intervention is needed in schools and in the community to improve the knowledge level [21]. Knowledge regarding fertility treatment from relevant education may encourage more women to visit fertility clinics with less stress and anxiety in Japan.

This study has some limitations. The survey was conducted in a single center located in an urban area where relatively high-income earners live. This setting may have influenced the lack of statistically significant changes in household income. In addition, because the survey was conducted shortly before and after the insurance coverage policy began, some patients (in the Before-coverage group) have known the details of the contents of insurance coverage and all the possible changes may not have been observed yet.

## Conclusions

In conclusion, more patients had an impression of infertility as a “disease” and preferred IVF as the first treatment option after health insurance coverage than before coverage. Furthermore, a lot of patients wanted to recommend oocyte freezing for their children despite a lack of insurance coverage. However, patients’ anticipated economic burden for treatment was not different between both groups.

This finding reveals the need to better disseminate information to patients in the future. Further studies are also needed in Japan since insurance coverage for fertility treatment began only recently, in April 2022.

## Appendices

**Table 3:** Anonymous questionnaire

Anonymous questionnaire on infertility treatment	
Q1: Have you received fertility treatment in another clinics?	1, First-time visit; 2, Examination/consultation only; 3, Visit with treatment
Q2: Please write down your annual household income.	（thousand yen）
Q3: Have you told others/colleagues in your workplace about this visit?	1, Not plan to do; 2, Plan to do; 3, Told
Q4: Do you know the differences between IUI and IVF?	1, Do not know; 2, Do not know much; 3, Know vague; 4, Know mostly; 5, Know
Q5: Do you know the differences between IVF and ICSI?	1, Do not know; 2, Do not know much; 3, Know vague; 4, Know mostly; 5, Know
Q6: Do you know about oocyte freezing?	1, Do not know; 2, Do not know much; 3, Know vague; 4, Know mostly; 5, Know
Q7: Which would you describe as infertile?	1, Condition; 2, Disease
Q8: Which applies to your current feelings?	1, Depressed; 2, Slightly depressed; 3, Cannot say either; 4, A little looking forward; 5, Looking forward
Q9: Would you consider IVF (egg retrieval, embryo transfer, etc.) as the first treatment?	1, Not considering; 2, Not considering much; 3, Cannot say either; 4, Little consideration; 5, Considering
Q10: Will you recommend the option of oocyte freezing for your daughter?	1, Will not recommend; 2, Unlikely to recommend much; 3, Cannot say either; 4. Would Consider; 5, Would Recommend
Q11: How much do you expect it will cost to perform one IVF procedure?	（thousand yen）
Q12: How many costs do you expect to incur until achieving pregnancy?	（thousand yen）
Q13: What is your impression of the costs in Q10 and Q11?	1, Inexpensive; 2. Low cost; 3, Reasonable; 4, Expensive; 5, Excessively expensive
Q14: Please provide any comments below regarding the insurance coverage of infertility treatment.	
※The Answer will not affect the treatment plan.	
